# Supplementary material for: Single-photon advantage in quantum cryptography beyond QKD
Source: Nat Commun. 2026 Feb 26;17:2074. doi: 10.1038/s41467-026-69995-9 (PMC12949056; doi:10.1038/s41467-026-69995-9)
Supplement: Supplementary file 1 — Supplementary Information [file 41467_2026_69995_MOESM1_ESM.pdf]

Supplementary Information for ”Single-Photon Advantage in Quantum Cryptography Beyond QKD”

Daniel A. Vajner[1,#], Koray Kaymazlar[1,#], Fenja Drauschke[2,#], Lucas Rickert[1], Martin v. Helversen[1], Hanqing Liu[3,4], Shulun Li[3,4], Haiqiao Ni[3,4], Zhichuan Niu[3,4], Anna Pappa[2], Tobias Heindel[1,5*]

[1] *Institute of Physics and Astronomy, Technical University of Berlin, 10623 Berlin, Germany*

[2] *Electrical Engineering and Computer Science Department, Technical University of Berlin, 10623 Berlin, Germany*

[3] *Key Laboratory of Optoelectronic Materials and Devices, Institute of Semiconductors, Chinese Academy of Sciences, Beijing, 100083, China*

[4] *Center of Materials Science and Optoelectronics Engineering, University of Chinese Academy of Sciences, Beijing, 100049, China*

[5] *Department for Quantum Technology, University of Münster, Heisenbergstraße 11, Münster, 48149, Germany*

[#] *These authors contributed equally.*

[*] *e-mail: tobias.heindel@uni-muenster.de*

Supplementary Note 1: Graphical discussion of honest abort probability

This note explores the behavior of the honest abort probability P_{AB} based on Supplementary Figure S1. At very low mean photon numbers μ, no events are detected and almost all detected events are due to dark counts, which means that in the honest case the parties would always abort. Increasing μ increases the amount of legitimate events, which reduces the need to abort the protocol. Similarly, when increasing the number of pulses K that Alice sends in each protocol run, the chance that a legitimate event is detected for a given μ increases which reduces the abort probability (Supplementary Figure S1 (right panel)). Additionally, detection errors also lead to honest aborts. A detection error is detected and leads to an abort in 1/2 of the cases. That is why the honest abort probability at high μ and K converges to $e/2$, which is also illustrated in Supplementary Figure S1. As the honest abort probability is not sensitive to the photon statistics, the main difference between Poisson-distributed sources like attenuated lasers and single photons lies in the cheating probabilities, as discussed in Supplementary Figure S2.

Supplementary Note 2: Graphical discussion on analytical cheating probability

This note complements Fig. 2a of the main text and further explores the behavior of Bob's cheating probability based on Supplementary Figure S2. Here, Bob's cheating probability is shown as a function of μ and K for both weak coherent pulses (WCPs) and a realistic single-photon source (SPS). Moreover, the classical cheating probability bound is shown and emphasizes the fact that the different photon distributions mainly affect the cheating probability and thus the parameter

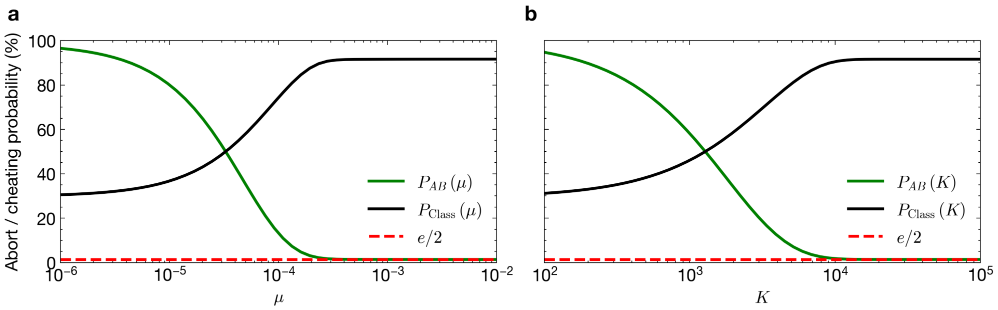

Supplementary Figure S1: Honest abort probability P_{AB} (green) and classical cheating probability P_{C} (black) versus μ (left panel) and K (right panel). The dashed constant red line represents the value of $\frac{e}{2} = 1.4\%$ to which the honest abort probability converges for large K and μ, respectively. Calculated with parameters from main text Table 1.

range in which a quantum advantage is possible. Also, as the green line is always below the blue line, the single photon advantage is present for all parameters.

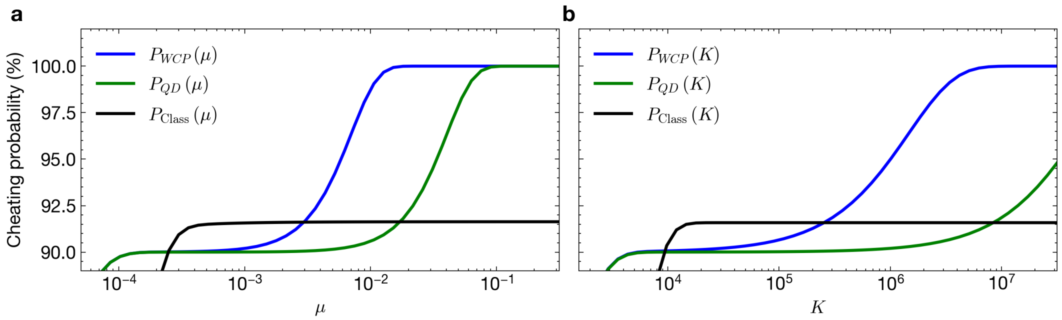

Supplementary Figure S2: Bob's cheating probabilities for WCPSs (blue) and QD source (green), together with classical cheating probability (black) versus μ (left panel) and K (right panel), calculated with parameters from main text Table 1.

Supplementary Note 3: Parameter Optimization

Figure S3 complements the Methods Section **Simulations for parameter optimization** in the main text by displaying simulation data for identifying the optimal parameter a. For the experimental parameters from Table 1 in the main text, a fair protocol is achieved for $a = 0.9$ (highlighted in red in Supplementary Figure S3b). Note how an increase in μ strongly increases Bob's cheating probability when attenuated laser pulses are used (blue lines), which requires a smaller a value to maintain fairness, and yields a higher cheating probability overall. The SPS (green lines) is less affected by the increase in μ due to less multi-photon events.

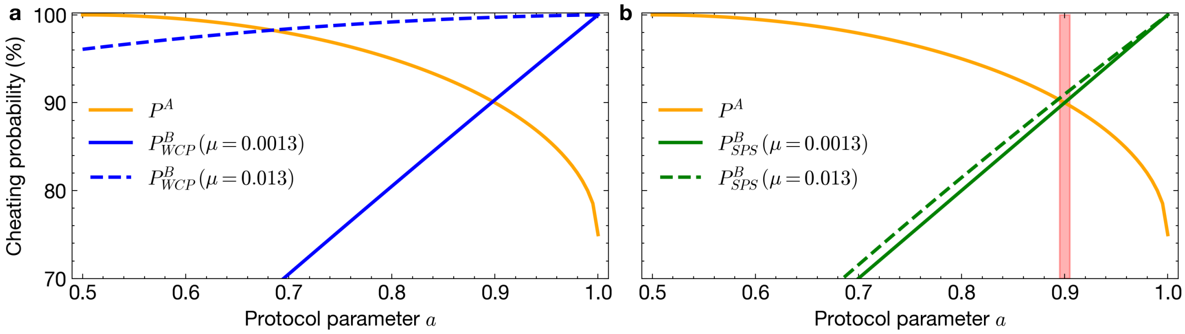

Supplementary Figure S3: Alice's cheating probability (orange) and Bob's cheating probability for (a) WCPs (blue, left panel) and (b) SPSs (green, right panel) versus state preparation parameter a with the parameters from main Table 1 and the mean photon number indicated in the legend. For the experimental parameters $P^A = P^B$ is achieved for $a = 0.9$ (highlighted in red in panel b).

Supplementary Note 4: Trade-off between single-photon and quantum advantage

This note also complements Fig. 2a of the main text and explains that we chose the optimal trade-off between the quantum advantage achieved relative to a classical protocol and a WCP implementation. As seen in Fig. 2a of the manuscript, one cannot simultaneously maximize the difference between quantum vs. classical and WCP vs. SPS. The maximum difference for the cheating probabilities between WCP and SPS occurs for parameters that no longer correspond to the maximum quantum advantage relative to the classical setting. This can be seen more clearly in Supplementary Figure S4 showing the differences in the respective cheating probabilities. While the SPS ensures a quantum advantage for a larger parameter range compared to the WCP source (green vs. blue curve), the SPS always performs better than the WCP source (red curve always larger than zero). Importantly, the maximum of the green curve (maximum quantum advantage) occurs for parameters different from the maximum of the red curve (maximum single photon advantage). In our work, we chose parameters that clearly yield a quantum advantage while simultaneously enabling a single-photon advantage. Additionally, choosing a smaller K means that the sequences are shorter and more coin flips can be performed per time.

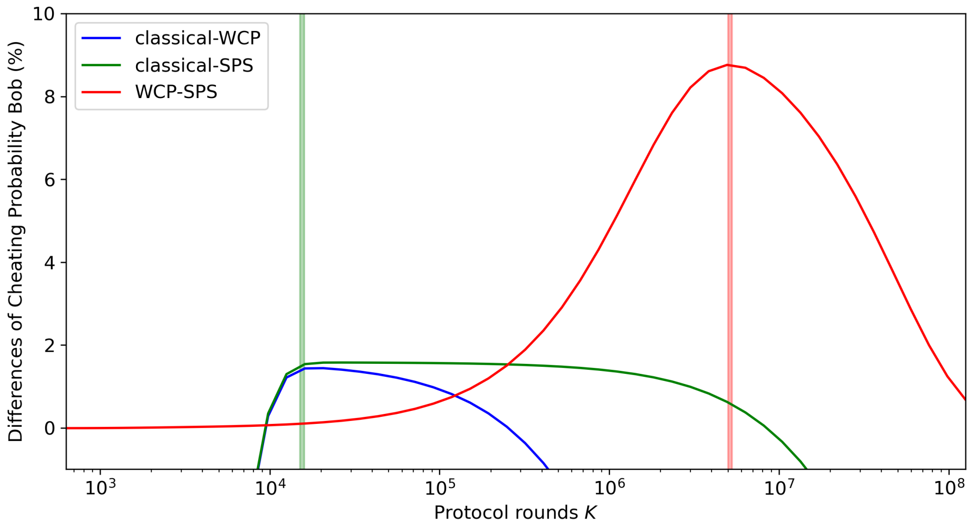

Supplementary Figure S4: Difference between classical and SPS cheating probability (green line), classical and WCP (blue line) and WCP and SPS (red line) to see that single photon advantage and quantum advantage do not happen for the same K for fixed $\mu = 0.0013$. The maximum quantum advantage is marked by the green vertical line, while the maximum single photon advantage is marked by the red vertical line.

Supplementary Note 5: Mapping from qubit to Stokes vector

In our experiment we employ polarization encoding and associate the state $|0\rangle$ and $|1\rangle$ with the linear polarizations $|H\rangle$ and $|V\rangle$. The corresponding polarization-encoded states are

$$\begin{aligned}
|\phi_{0,0}\rangle &= \sqrt{a}|H\rangle + \sqrt{1-a}|V\rangle \\
|\phi_{0,1}\rangle &= \sqrt{1-a}|H\rangle - \sqrt{a}|V\rangle \\
|\phi_{1,0}\rangle &= \sqrt{a}|H\rangle - \sqrt{1-a}|V\rangle \\
|\phi_{1,1}\rangle &= \sqrt{1-a}|H\rangle + \sqrt{a}|V\rangle \quad .
\end{aligned} \tag{1}$$

Note that a certain qubit superposition does not correspond to a certain Stokes vector superposition. In other words the qubit $|\psi\rangle = (|0\rangle + |1\rangle)/\sqrt{2}$ which we want to associate with the polarization state $|D\rangle = (|H\rangle + |V\rangle)/\sqrt{2}$ is not obtained by the polarization Stokes vector

$$\begin{aligned}
\left(\vec{S}_H + \vec{S}_V\right)/\sqrt{2} &= \left((1,1,0,0)^T + (1,-1,0,0)^T\right)/\sqrt{2} \\
&\neq \vec{S}_D = (1,0,1,0)^T \quad .
\end{aligned}$$

Such a direct mapping of superpositions on the Bloch sphere to superpositions on the Poincare sphere is also not possible, since the the polarization Stokes vector entries are real numbers, while the Bloch sphere allows for complex superpositions.

In order to prepare these states experimentally, we are interested in a general formulation of the Stokes vectors for these states as a function of the state preparation parameter a. By using the classical definitions of the Stokes vector and inserting the projection probabilities $P_k = |\langle\phi|\phi_k\rangle|^2$ into the standard $k = \{H, V, D, A, R, L\}$ polarization basis states, we can write the Stokes vector

components as

$$
\begin{aligned}
S_1 &= P_H + P_V \\
S_2 &= P_H - P_V \\
S_3 &= P_D - P_A = P_{(H+V)/\sqrt{2}} - P_{(H-V)/\sqrt{2}} \\
S_4 &= P_R - P_L = P_{(H+iV)/\sqrt{2}} - P_{(H-iV)/\sqrt{2}} \quad .
\end{aligned}
$$

Thus, we can project each of the four general input states (cf. equation 1 from main text) in the correct polarization basis to obtain the entries of the Stokes vector as a function of a as

$$
\begin{aligned}
\vec{S}_{0,0} &= \begin{bmatrix} 1 \\ 2a-1 \\ 2\sqrt{a(1-a)} \\ 0 \end{bmatrix}, \vec{S}_{0,1} = \begin{bmatrix} 1 \\ 1-2a \\ -2\sqrt{a(1-a)} \\ 0 \end{bmatrix}, \\
\vec{S}_{1,0} &= \begin{bmatrix} 1 \\ 1-2a \\ 2\sqrt{a(1-a)} \\ 0 \end{bmatrix}, \vec{S}_{1,1} = \begin{bmatrix} 1 \\ 2a-1 \\ -2\sqrt{a(1-a)} \\ 0 \end{bmatrix}.
\end{aligned}
$$

Knowing the Stokes parameters, we can prepare the states in the experiment using wave-plates and align the detection basis to these expected protocol states.

Supplementary Note 6: Manchester Coding

Supplementary Figure S5 complements Methods section **Four-state Manchester coding** in the main text by showing extended data confirming the suppression of voltage level drifts when Manchester Coding is applied.

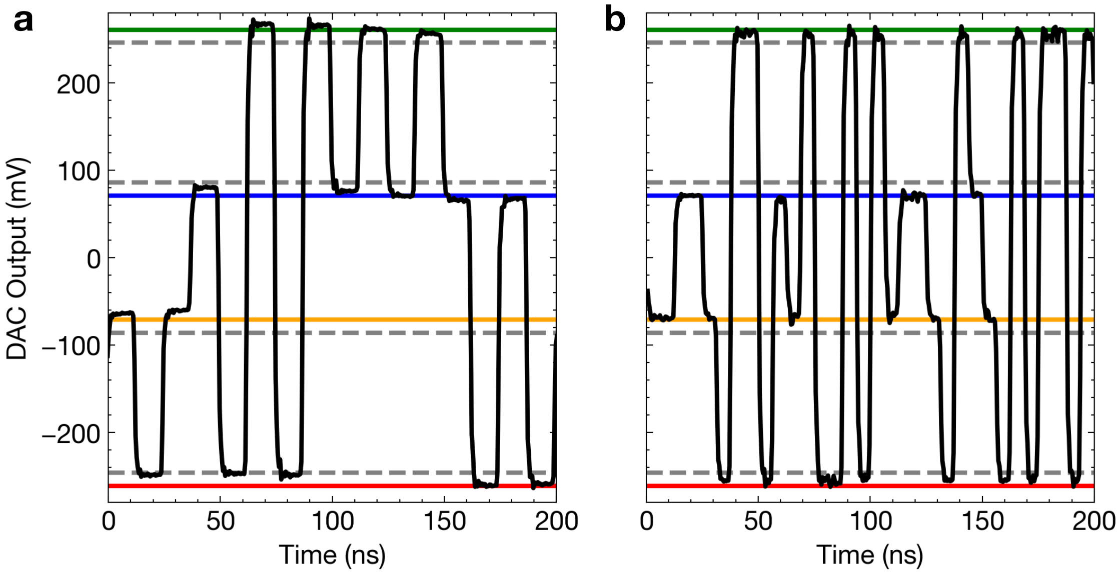

Supplementary Figure S5: Output voltage of the DAC stage measured for a sequence of random voltage levels targeting the four QSCF states (solid lines). The voltage levels corresponding to the standard BB84 states are indicated as reference (dashed lines). (a) Direct encoding of voltage levels at 80 MHz optical- and electronic-clock rate results in voltage level drifts increasing the QBER. (b) Applying the advanced encoding scheme with additional orthogonal voltage levels by doubling the internal clock rate of the AWG to 160 MHz effectively suppresses the voltage drift.

Supplementary Note 7: Offset Calibration

Supplementary Figure S6 complements the Methods Section **Data Analysis** in the main text by displaying extended data for determining the relative shift between the QCF state symbols sent and detected during the execution of the protocol. Here, the correct offset between both lists is found to be 16 pulses as indicated by the clear dip in Supplementary Figure S6, corresponding to a temporal shift of $\approx 200\,\text{ns}$. For all other combinations, the curve converges to the value of 1.25 expected for the mean difference between two random numbers from the sets $[1, 2, 3, 4]$.

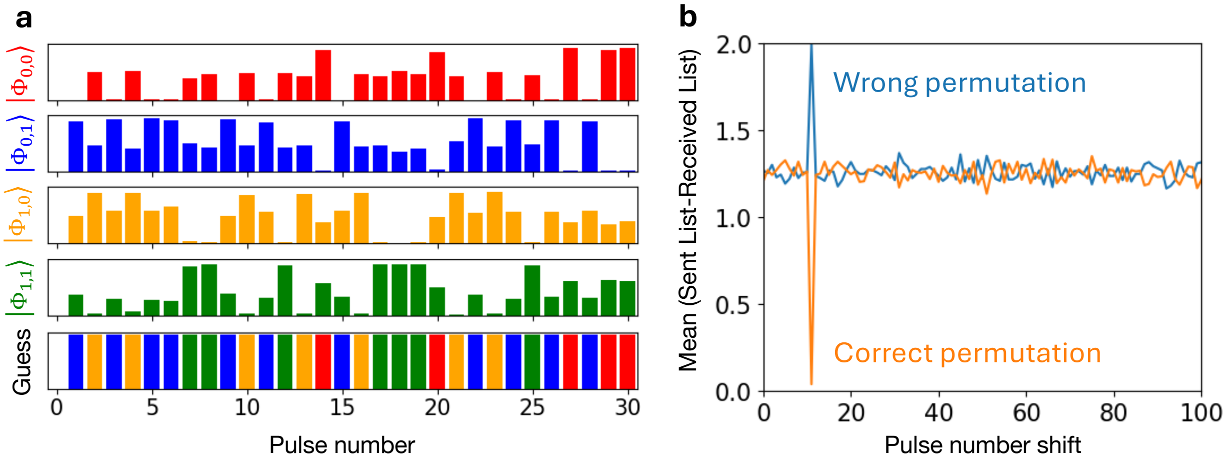

Supplementary Figure S6: (a) Determining the shift between sent and detected state lists by summing over many repeating random sequences and guessing the detected state. The colors correspond to the different detected states and the lowest panel indicates the guessed state. (b) Comparing sent and received lists yielding a relative shift of 16 pulses.

i	$P(A_i)$	$P(b' \mid A_i)$
1	p_0^K	$= 0.5$
2	$(p_0 + p_1)^K - p_0^K$	$\leqslant a$
3	$Kp_2 p_0^{K-1}$	$= a$
4	$Kp_2((p_0 + p_1)^{K-1} - p_0^{K-1})$	$= -2a^2 + 4a - 1$

Supplementary Table 1: Probabilities $P(A_i)$ and cheating probabilities $P(b' \mid A_i)$ entering eq. 4 in the main text.

$P = \| \langle \phi_{i,j} \| \phi_{k,l} \rangle \|^2$	$\|\phi_{0,0}\rangle$	$\|\phi_{0,1}\rangle$	$\|\phi_{1,0}\rangle$	$\|\phi_{1,1}\rangle$
$\langle \phi_{0,0} \|$	1	0	$4a(1-a)$	$(2a-1)^2$
$\langle \phi_{0,1} \|$	0	1	$(2a-1)^2$	$4a(1-a)$
$\langle \phi_{1,0} \|$	$4a(1-a)$	$(2a-1)^2$	1	0
$\langle \phi_{1,1} \|$	$(2a-1)^2$	$4a(1-a)$	0	1

Supplementary Table 2: Probability that a prepared state is projected into one of the four basis states. Using this table, the theoretically expected input-output matrix in main Figure 5c was calculated.
